# The Hypodermic Use of Cocaine

**Published:** 1895-04

**Authors:** H. B. Hinman

**Affiliations:** W. of M.


### The Hypodermic Use of Cocaine.

BY H. B. HINMAN, W. OF M.

Any method of preventing or alleviating pain in the extraction of teeth has always been hailed by the dental profession and its patrons with rejoicing.

Of all the local anæsthetics which have come into use within the last decade, there is none which is so powerful, or which has attained such universal use and wide-spread popularity as cocaine, or, more properly speaking, the hydrochlorate of cocaine, the alkaloid itself not being used for this purpose because of its slight degree of solubility.

When the drug first came into prominence as a local anæsthetic, it was used in much stronger solutions than it is at the present day, many employing as high as a 20-per-cent. solution. It is now almost universally conceded, however, that better results, with far less danger accompanying their use, are obtained by much weaker preparations, say a 2-per-cent. solution.

Cocaine forms the basis of nearly all the local anæsthetics on the market to-day, being combined in these preparations with other drugs, some of which act as adjuvants, and others as correctives and diluents.

One of the best formulæ which we have to-day for the administration of cocaine hypodermically, is that furnished us by Dr. Hoff, and is as follows:

℞. Cocaine Hydrochlorate......$\frac{1}{2}$ grain.

Morphine Sulphate..........$\frac{1}{4}$ grain.

Atrophine Sulphate.........$\frac{1}{100}$ grain.

Distilled Water....... .... ..25 M.

When compounded in these proportions, we have a full dose of a two-per-cent. solution, in which the morphine acts as a corrective to the cocaine, and the atrophine serves the double purpose of being a corrective to both the cocaine and the morphine.

Cocaine preparations should be renewed at least every three or four days, and preferable every day, as, after being kept a short time, a mould forms in the solution, which so chemically changes the cocaine that it will cause sloughing of the gums.

Care should always be taken in the selection of the patient, as weak and nervous persons, and those having diseases of the heart and lungs, are particularly subject to the toxic effects of the drug; and it should be used very gradually, if at all, in these cases.

The time of day also has a marked bearing on the subject, as the patient bears the administration of the drug much better early in the day, and after a hearty meal, than later on, when tired out and when several hours have elapsed since the taking of nourishment.

The toxic effects of cocaine are exhibited by an embarrassment of the respiration and circulation. Respiration becomes slow and shallow, and the pulse small, rapid and intermittent, and after a time, if the toxic effects be sufficient, they may cease altogether.

Cocaine should never be administered without having the proper antidotes and resuscitants at hand for use in any emergency that may occur.

Ammonia and nitrate of amyl are both given by inhalation for the purpose of stimulating the respiration and the heart. Opium and chloral are also administered, but in extreme cases a hypodermic injection of aromatic spirits of ammonia or sulphate of strychnia, together with artificial respiration, are the most efficacious.

It is well, when the patient shows signs of nervousness, to administer a little brandy, or ten to fifteen drops of aromatic spirits of ammonia well diluted with water, a few minutes before the operation, thus allaying the nervousness and insuring a strong, steady pulse throughout the course of the drug's action.

There are many forms of the hypodermic syringe on the market to-day which are equally good—the two most important points in regard to their construction being that they are easy of manipulation and readily sterilized.

The points of the needles, as bought at the depots, are too sharp to work satisfactorily, as they are apt to become bent, and they are also troublesome, as they catch in the alveolar process very readily. It is always best to round them off somewhat on an oilstone before using.

The needle should be inserted a short distance from the neck of the tooth, and pressed slowly but firmly toward the apex of the root. After passing into the tissues for a short distance, a drop or two of the solution should be slowly injected, and the needle then pressed forward a little further and a small quantity more forced into the tissues in a similar manner.

The amount and rapidity of the injection should be governed by the density of the tissues, as those which are very dense will take up less of the solution and require a smaller amount to anæsthetize them than where they are more loosely arranged.

The needle should not be withdrawn for about a minute after the injection is made, and then the finger should be placed over the puncture for a short time, to prevent the exudation of the cocaine solution.

Care must also be taken not to inject into an abscess cavity, as the fluid will then escape into the mouth, and is apt to paralyze the muscles of the fauces and the other accessory muscles of respiration there.

If the needle penetrates one of these abscess cavities, it should be at once removed and inserted at another point.

Two injections of from two to five drops each, of a two-per-cent. solution, one on each side of the tooth, are usually sufficient to produce complete insensibility to the pain of the operation. Many of the cases of sloughing of the gums, attributed to cocaine, are due to septic material introduced by unclean forceps, and would have occurred had the cocaine not been used.

We cannot be too careful in attending to the proper sterilization of the needle, lancet and forceps.

If the mouth be rinsed out freely with warm water for some minutes after the operation, it will tend to prolong the bleeding, and thus relieve the gorging of the blood vessels caused by their relaxation, after the over-stimulation from the use of the drug.

When these precautions are taken and strong solutions are not used, the use of cocaine will seldom be accompanied by sloughing of the gums.

